# Multimodal learning on heterogeneous subgraphs and LLMs representation for MHC-peptide binding affinity prediction

**DOI:** 10.1186/s12859-026-06407-1

**Published:** 2026-02-28

**Authors:** Ruimeng Li, Ying Wang, Haozhou Li, Biyi Zhou, Qinke Peng

**Affiliations:** Faculty of Electronic and Information Engineering, The Systems Engineering Institute, Xi’an Jiaotong University, Xi’an, 710049 China

**Keywords:** Heterogeneous graph, Cross-attention, Edge-induced subgraph extract, GCN, Contrastive learning

## Abstract

Accurate prediction of MHC-peptide binding affinity remains a challenge for immunotherapeutic development. Existing methods struggle to jointly model functional semantics of polymorphic residues, evolutionary conservation constraints, and structural dynamic. We propose the Contrast learning-based Multi-feature Heterogeneous Subgraph model (CMHS) with sequence and structural representation. For sequence representation, we introduce LoRA fine-tuning to obtain the MHC-exclusive sequence representation from ESM2, then jointly BLOSUM50 to capture long-range functional dependencies and evolutionarily conserved residues. For structural representation, we use the biophysics-guided heterogeneous graph network. Constructing an MHC-peptide graph with a novel trainable Gaussian noise layer guided by crystallographic B-factors to dynamically simulate electron density uncertainty, coupled with a three-stage message-passing framework with subgraph aggregation, subgraph extraction and heterogeneous. Finally, to align sequence and graph representation spaces, we use contrastive learning to obtain a more comprehensive representation and to enhance the ability of model prediction. Evaluations on 16 HLA allele benchmarks show average SRCC improvements of 8.7$\%$, with improvements of average AUC of 7.6$\%$. This work establishes a new paradigm for predicting hypervariable immune interactions. The corresponding code can be founded in github.

## Introduction

The Major Histocompatibility Complex (MHC) mediates adaptive immunity by presenting peptide fragments on cell surfaces for T-cell recognition [1]. However, its extreme genetic polymorphism encompassing over 20,000 documented alleles poses a significant challenge: experimental determination of binding affinities is infeasible at this scale due to combinatorial explosion [2–5].

Computational methods for predicting MHC-peptide binding affinities are generally divided into two categories: allele-specific and pan-allele methods [2]. While allele-specific models train predictors for each allele, they fail to generalize across the polymorphic allele [4–6]. Pan-allele methods (e.g., the NetMHCpan series) leverage shared binding patterns. For instance, NetMHCpan1.0 to NetMHCpan3.0 are sequence-based tools using artificial neural networks [7, 8]. To enhance performance, NetMHCpan4.0 integrated mass spectrometry (MS) data to capture in vivo peptide repertoires [9, 10]. Other models, such as MHCflurry, extract features via fully connected layers [11]. However, MS data suffers from systematic deviations due to limitations in proteolytic efficiency, ionization, and detection sensitivity, and only a small subset of peptides have experimentally validated binding specificities. Moreover, MS data lacks structural information and evolutionarily conserved signals [12]. Thus, building a universal, robust, and interpretable MHC-peptide binding model solely on MS data remains challenging. Many models employ biophysical and model-based representations for sequence data. However, current representation schemes face limitations. Matrix-based encodings, such as BLOSUM50 in ACME capture residue substitution constraints but are processed by CNNs without explicitly modeling allele-specific functional semantics or spatial conformations [13]. Similarly, MHCSeqNet uses one-hot encoding combined with skip-gram methods (designed for natural language processing) to learn amino acid correlations and incorporates one-hot allele representations [14]. These approaches fail to capture the spatial topology of the binding interface.

Pseudo-sequence methods represent MHC-peptide binding patterns by selecting residues experimentally verified to bind peptides. However, compressing 3D binding sites into 1D residue indexes disrupts spatial relationships [15–17]. While methods like RPEMHC [18] and ConvNeXt-MHC [19] introduce residue-pair encodings to capture spatial interactions, they are confined to local contacts and neglect long-range and indirect contributions [20, 21]. Moreover, by focusing on single amino acids, they ignore the broader context within the MHC. Crucially, all static representations fail to model peptide-induced conformational changes, introducing systematic bias in flexible regions.

Recently, Large Language Models (LLMs) based on the Transformer framework have been applied to learn richer semantic representations, capturing functional and structural features [22, 23]. For example, pMHChat [12, 24] integrates ESM-MSA-1b and ESM-2 embeddings and uses BiLSTM to capture long-range dependencies in MHC sequences, addressing the limitations of traditional LSTMs in modeling remote associations of polymorphic residues. It also employs hypergraph neural networks for anchor prediction. Despite these advances, LLMs exhibit limitations: they are insufficiently sensitive to single-point variations (common in MHC alleles), and direct fusion of sequence embeddings with structural features creates representation space discordance, propagating learning bias.

To address these challenges, we propose CMHS (Contrastive Multi-feature Heterogeneous Subgraph), a deep learning framework for predicting MHC-peptide binding affinity. CMHS introduces three key innovations: To overcome the limitations of matrix-based representations, we jointly fine-tune ESM-2 on MHC sequences with BLOSUM50 constraints, synergizing conserved residue signals and long-range dependencies. To address the lack of structural data and spatial topology loss, we employ ESM-3 to predict MHC structures, modeling inter-domain couplings. We then construct MHC-peptide heterogeneous graphs incorporating a B-factor-guided trainable Gaussian noise layer to dynamically simulate electron density uncertainty. To resolve representation space discordance, we use contrastive learning to align sequence and graph representations, eliminating modality bias.

## Methods

### Preliminaries

**Fig. 1:**
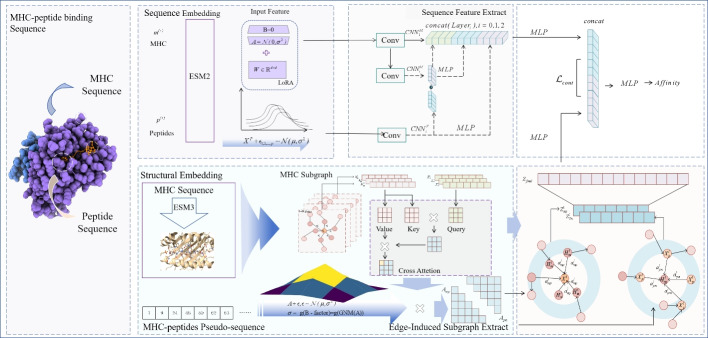
Overall framework of CMHS model

Denote ${M} = \{{m^{\left( 1 \right) }},{m^{\left( 2 \right) }},...,{m^{\left( n \right) }}\}$ as MHC samples, ${P} = \{{p^{\left( 1 \right) }},{p^{\left( 2 \right) }},...,{p^{\left( q \right) }}\}$ as peptides samples. Given $\left\{ { \left( {{m^{\left( i \right) }},{p^{\left( i \right) }}} \right) ,{a^{\left( i \right) }}}\right\}$ where $\left( {{m^{\left( i \right) }},{p^{\left( i \right) }}} \right)$ is a MHC-peptide pair and $a^{\left( i \right) }$ is the corresponding afinity value. The main goal is to design a system that takes the MHC-peptide pair as the input to predict the affinity values as shown in the Fig. 1.

### The embedding of MHC and peptides sequences

#### BLOSUM50 embedding

Firstly, we use BLOSUM50 to represent MHC and peptides. The BLOSUM matrices is one of the most useful representation methods calculated in large protein dataset and is able to evaluate the probability of replacement of each AA to deduce the physical properties [7, 8]. Considering the length of the peptide sequences ranges from 9 to 12, we use an encoded method to unify the input length [13].

#### LLM-based

BLOSUM50 is a static matrix and cannot capture context information. For instance, the function of an amino acid may vary at different positions, but BLOSUM50 only considers pairing substitution and does not take the surrounding environment into account. Second, it may not be able to handle polymorphic regions in the MHC that are highly variable but structurally critical. In addition, BLOSUM50 is based on distant homologous sequences and may not be applicable to rapidly evolving gene families like MHC. Large language models are widely used in protein representation due to their ability of context-aware dynamic representation. MHC molecules are highly polymorphic, and there are key functional sites (such as peptide-binding groove pockets) in the sequence. General pre-trained models may not be able to focus on these key regions or capture the functional implications of subtle differences among alleles. We use a large model representation combined with fine-tuning to characterize MHC sequences. ESM2, a transformer-based protein language model at scales from 8 million parameters up to 15 billion parameters, is one of the widely used protein Masked Language Model (MLM)[24]. To reduce the fine-tuning parameters, we use the LoRA for ESM2 fine-tuning [25].

The BLOSUM50 embeddings of the MHC sequence is denoted as ${X^{MB}} = \{{x_1^{MB}},{x_2^{MB}},...,{x_n^{MB}}\}$, $x_i^{MB} \in {\mathbb {R}^{l \times d}}$ represent the $i-th$ MHC sequence with length of *l*. The BLOSUM50 embeddings of the peptide sequence is denoted as ${X^{PB}} = \{ {x_1^{PB}},{x_2^{PB}},...,{x_q^{PB}}\}$, $x_i^{PB}\in {\mathbb {R}^{p \times d}}$ represent the $i-th$ peptides sequence with length of *p*.

The ESM2 of the MHC sequence is denoted as ${X^{ME}} = \{{x_1^{ME}},{x_2^{ME}},...,{x_n^{ME}}\}$, $x_i^{ME} \in {\mathbb {R}^{l \times e}}$ represent the $i-th$ MHC sequence with length of *l*, $e=320$. Let $\phi$ be the frozen pretrained ESM2 model with fine-tuning parameters $\theta$. For output MHC sequence $x_i^{ME}$:

1 $$\begin{aligned} x_i^{ME} = \phi (p{}^{(i)};\theta ) \end{aligned}$$

where $x_i^{ME} \in \mathbb {R}^{l \times e}$ is the output embedding. For any weight matrix $W \in \mathbb {R}^{d_a \times d_b}$ in ESM2 in Q/K/V projections, LoRA decomposes updates into low-rank matrices:

2 $$\begin{aligned} {W_{new}} = W + \Delta W = W + B{A^T} \end{aligned}$$

where $\textbf{A} \in \mathbb {R}^{d_b \times r}$ and $\textbf{B} \in \mathbb {R}^{d_a \times r}$ is trainable parameters, $r \ll \min (d_a, d_b)$ is the LoRA rank which is hyperparameter. For input *M* to a self-attention layer:

3 $$\begin{aligned} \begin{gathered} Q = {M}({W_Q} + {B_Q}A_Q^T) \\ K = {M}({W_K} + {B_K}A_K^T) \\ V = {M}({W_V} + {B_V}A_V^T) \\ \end{gathered} \end{aligned}$$

Only ${\textbf{B}_Q, \textbf{A}_Q, \textbf{B}_K, \textbf{A}_K, \textbf{B}_V, \textbf{A}_V}$ are updated during fine-tuning.

The final output of embedding of MHC sequence can be denoted as ${X^M \in \mathbb {R}^{m\times {d+e}} }$:

4 $$\begin{aligned} X^{M} = concat(X^{ME},X^{MB}) \end{aligned}$$

The ESM2 embeddings of the peptide sequence is denoted as ${X^{PE}} = \{ {x_1^{PE}},{x_2^{PE}},...,{x_q^{PE}}\}$, $x_i^{PE}\in {\mathbb {R}^{p \times e}}$ represent the $i-th$ peptides sequence with length of *p*. Due to the short length of peptides, they are directly characterized using ESM2. Let $\phi$ be the frozen pretrained ESM2 model with parameters $\theta$. For output peptides sequence $x_i^{PE}$:

5 $$\begin{aligned} x_i^{PE} = \phi (p{}^{(i)}) \end{aligned}$$

where $x_i^{PE} \in \mathbb {R}^{p \times e}$ is the output embedding with frozen ESM2.

Same, the final output of embedding of MHC sequence can be denoted as ${X^P \in \mathbb {R}^{p\times {d+e}} }$:

6 $$\begin{aligned} X^{P} = concat(X^{PE},X^{PB}) \end{aligned}$$

### Multi-level sequence feature pyramid-module

To simulate the recognition mechanism: MHC-peptide binding relies on both global structural complementarity and local matching of key anchor residues, we use a layer-by-layer fusion approach to extract the sequence features introduced in [13].

To simulate natural amino acid mutations in peptide-MHC interactions and enhances generalization to unseen alleles through controlled perturbation, we introduce the trainable knowledge-based learnable Gaussian noise module. This module enhances model robustness by injecting learnable Gaussian noise into the embedding space. Unlike traditional fixed noise, both the mean ($\mu$) and standard deviation ($\sigma$) are dynamically generated by neural networks conditioned on input features. Specifically, For input of peptides embedding feature ${X^P \in \mathbb {R}^{p\times {d+e}} }$:

7 $$\begin{aligned} {\tilde{X^P}} = {X^P} + \epsilon \odot I,\epsilon _{1,2,...,p} \sim \mathcal {N}(\mu ,{\sigma ^2}) \end{aligned}$$

where, $\epsilon$ is the inject gaussian noise, $\textbf{I}$ is the dimensionally adapted unit tensor, $\sigma = \exp (\log \sigma )$ and $\mu$is the trainable standard deviation parameter.

Then we used two layers of CNN to extract MHC sequence feature and a single CNN layer to extract the peptides sequence feature based on the different length. We have established feature link modules at each layer to obtain features of different dimensions. Denoted the *i*-th CNN layer for MHC as $CNN^M_i, i=1,2$, the $CNN^P$ for peptide, the module can be defined as:

8 $$\begin{aligned} \begin{gathered} Layer_0=concat(X^M,X^P)\\ Layer_i=concat(CNN^M_i,CNN^P)\\ \end{gathered} \end{aligned}$$

Final output of Multi-Level Sequence Feature Pyramid-module is:

9 $$\begin{aligned} S_{final}=concat(Layer_i), i=0,1,2 \end{aligned}$$

### The embedding of MHC and peptides structure

The real structure data for MHC and peptides given by experiment is limitation compared with sequences data. Due to the efficiency of structure prediction model in protein domain, we use ESM3 model to predict the structure data for each MHC atom. To transform atomic-level information into residue-level representations that are more suitable for graph neural network processing, we calculate the 3-dimensional geometric center for each amino acid residue, based on the coordinates of its relevant atoms. This centroid coordinate represents the position of the residue.

The binding pratten we used the pseudo-sequence proposed by [7], which contain the position of binding sites obtained from biological experiments. We defined the MHC-peptides binding systems as heterogeneous graph $\mathcal {G}=(\mathcal {V},\mathcal {E},\mathcal {A},\mathcal {R}, \tau , \phi )$, thereinto, $\mathcal {V} = \mathcal {V}_M \cup \mathcal {V}_P$, $\mathcal {V}_M$ is the nodes in MHC, $\mathcal {V}_P$ is the nodes in peptide; $\mathcal {A} = {MHC,Peptide}$ is the type of nodes; $\mathcal {R} = {MHC-MHC, MHC-Peptide, Peptide-MHC}$ shows the type of edges, also, we have:

10 $$\begin{aligned} \tau (v) = \left\{ {\begin{array}{*{20}{c}} {MHC\quad \forall v \in {\mathcal {V}_M}} \\ {Peptide\quad \forall v \in {\mathcal {V}_P}} \end{array}} \right. \end{aligned}$$

11 $$\begin{aligned} \phi (e) = \left\{ {\begin{array}{*{20}{c}} {MHC - MHC\quad if({\nu _i},{\nu _j}) \in {\mathcal {E}_{mm}}} \\ {MHC - Peptide\quad if({\nu _i},{\nu _j}) \in {\mathcal {E}_{mp}}} \\ {Peptide - MHC\quad if({\nu _i},{\nu _j}) \in {\mathcal {E}_{pm}}} \end{array}} \right. \end{aligned}$$

where $\mathcal {E}_{mm} \subseteq \mathcal {V}_m \times \mathcal {V}_m$ represent the interior edges in MHC. $\mathcal {E}_{mp} \subseteq \mathcal {V}_m \times \mathcal {V}_p$ represent the outside edges from MHC to peptide, $\mathcal {E}_{pm} \subseteq \mathcal {V}_p \times \mathcal {V}_m$ represent the outside edges from peptide to MHC.

The definition of $\mathcal {E}_{mm}$ based on the result from ESM3. Specifically, for each $C_{\alpha }$ atom, calculate the Euclidean distance between it and other $C_{\alpha }$ atoms, and then generate an adjacency matrix based on the threshold we defined as 8 Å (if the distance is less than the threshold, the corresponding position is 1; otherwise, it is 0). Note that the adjacency matrix should be symmetrical and have a diagonal of 0. The prediction structure data often introduce bias and noise, limiting effectiveness [26].

Assumed that the deformation field is constant locally while docking, the density around the molecule moves in a similar way. The deformation in the continuous molecular docking can be described as the displacement of the Gaussian center [27]. Here, we introduce trainable 2-dimensional Gaussian noise in $\mathcal {E}_{mp}$ to $\mathcal {E}_{pm}$ simulate the flexibility of side chain, and enhance the robustness. Specifically:

12 $$\begin{aligned} {\tilde{A}} = A + \epsilon ,\epsilon \sim \mathcal {N}(\mu ,{\sigma ^2}) \end{aligned}$$

$\epsilon$is implemented through trainable parameters with $\mu$ and $\sigma$. The $\mu$ parameter represents the distance distribution between the $C_{\alpha }$ atom and the actual anchor positioning, calculate by:

13 $$\begin{aligned} \mu =f(A) \end{aligned}$$

$\sigma$ parameter fuses the crystallographic B factor to simulate the uncertainty of the electron density cloud, calculate by:

14 $$\begin{aligned} \sigma = g(B - factor)=g(GNM(A)) \end{aligned}$$

*g* is the transform function, introduced by the relationship between factor-B and the standard deviation of atomic shift in crystallography as inverse. Due to the nonlinear threshold response of the flexible change of residues, which shows a tendency towards saturation at positions with large distances, we select the sigmoid form to model the relationship between sigma and the B-factor.

15 $$\begin{aligned} g(B) = {\sigma _{\min }} + ({\sigma _{\max }} - {\sigma _{\min }}) \times \frac{1}{{1 + {e^{ - k(B - {B_m})}}}} \end{aligned}$$

here, $\sigma _{\min }$, $\sigma _{\max }$, $B_m$ and *k* is the hyperparameters. *GNM* denote the Gaussian Network Model (GNM), introduced in [28] is able to predict the B-factor:

16 $$\begin{aligned} {\Gamma _{ij}} = \left\{ \begin{aligned}&- 1\quad \quad&if\;i \ne j\;and\;{A_{ij}} > 0 \\&0\quad \quad&if\;i \ne j\;and\;{A_{ij}} < 0 \\&- \sum \nolimits _{k \ne i} {{\Gamma _{ik}}} {\hspace{1.0pt}} \;&if\;i = j \end{aligned} \right. \end{aligned}$$

17 $$\begin{aligned} B_i=\frac{{{k_B}T}}{\gamma }{[{\Gamma ^{ - 1}}]_{ii}} \end{aligned}$$

where $k_B$ is the Boltzmann constant, *T* denote the temperature constant and $\gamma$ is the force constant.

### Subgraph extraction-based heterogeneous graphs

MHC structures and their interactions with peptides is the hierarchical organization. To decouple intrinsic MHC structural representation learning from peptide-induced binding interactions, while enabling effective information exchange between the two components, we formulate the binding system as a subgraph extraction-based heterogeneous graph.

Specifically, the MHC molecule is first represented as an independent subgraph, where intra-MHC residue interactions are modeled using a graph attention network (GAT) to capture its structural topology and long-range dependencies. Subsequently, MHC-peptide interactions are incorporated by introducing a cross-attention mechanism between the MHC subgraph and the peptide residues. The resulting cross-attention scores are used to construct an interaction-aware adjacency matrix, which explicitly encodes peptide-MHC coupling. Based on this adjacency structure, a graph convolutional network (GCN) is applied to jointly propagate information across the peptide and MHC subgraph, enabling effective integration of structural context and binding-induced perturbations. The final graph representation is then used to predict peptide-MHC binding affinity.

#### Prior attention-guided MHC subgraph module

When MHC interacts with small peptides, it is often the interaction of microenvironments with adjacent spatial structures. To obtain the characteristics of adjacent nodes within the spatial structure of MHC, we first use the Graph Attention Network (GAT) to calculate the MHC subgraph under the heterogeneous graph framework.

For this part, we directly use the BLOSUM50 encoding result as the node representation to reduce the network depth of the graph part. For each MHC node $v_m \in \mathcal {V}_m$, calculate its interaction attention:

18 $$\begin{aligned} H_m = GAT(X^{MB},{A_{mm}}) \end{aligned}$$

19 $$\begin{aligned} A_{mm}^{ij} = \left\{ {\begin{aligned}&Adjacency({v_i},{v_j})&\tau ({v_i}) = \tau ({v_i}) = MHC \\&0&otherwise \end{aligned}} \right. \end{aligned}$$

$H_m^{(l + 1)}$ is the output of GAT layer, and $Adjacency({v_i},{v_j})$ is the adjacency relationship calculated from the structure predicted by ESM3. The implementation of the GAT layer is:

20 $$\begin{aligned} h_i^{(l + 1)} = \sum \limits _{k = 1}^K {\sigma \left(\sum \limits _{j \in {\mathcal {N}_i}} {\alpha _{ij}^k{W^k}h_j^{(l)}} \right)} \end{aligned}$$

$\alpha _{ij}^k$is the attention weight of the *K*-th attention head:

21 $$\begin{aligned} {\alpha _{ij}} = \frac{{\exp (Leaky\operatorname {Re} LU(a([W{h_i}||W{h_j}])))}}{{\sum \nolimits _{k \in {\mathcal {N}_i}} {\exp (Leaky\operatorname {Re} LU(a([W{h_i}||W{h_k}])))} }} \end{aligned}$$

#### Cross-attention-based edge-induced subgraph extraction module

Our heterogeneous graph is defined as a graph containing both MHC and peptide nodes. Now, we need to extract the subgraphs in the MHC-peptide and peptide-MHC relationships. The subgraphs include all nodes in peptide and the top-k nodes in MHC that are closest to the peptide node. Based on the definitions extracted from the Edge-Induced subgraphs in the graph, we provide the definitions: Given a heterogeneous graph $G = (\mathcal {V}, \mathcal {E})$ with:

- Vertex set $\mathcal {V} = \mathcal {V}_M \cup \mathcal {V}_P$ ($\mathcal {V}_M$: MHC nodes, $\mathcal {V}_P$: peptide nodes)
- Edge set $\mathcal {E} \subseteq \mathcal {V}_M\times \mathcal {V}_P$ (MHC-peptide edges only)

Let $\textrm{score}: \mathcal {E} \rightarrow \mathbb {R}$ be an edge weighting function and $k \in \mathbb {N}^+$ a predefined constant. The subgraph $G_{\textrm{sub}} = (\mathcal {V}_{\textrm{sub}}, \mathcal {E}_{\textrm{sub}})$ is constructed as:

$$\begin{aligned} 1.&\ \text {Preserve all peptide nodes: } \mathcal {V}_p \subseteq \mathcal {V}_{\textrm{sub}} \\ 2.&\ \text {For each } p \in \mathcal {V}_p \text {, select top-}k \text { incident edges:} \\&\quad \mathcal {E}_p = \left\{ e \in \mathcal {E} \mid e = (m, p) \wedge m \in V_m \wedge \textrm{rank}_p(e) \le k \right\} \\ 3.&\ \text {Vertex set: } \\&\quad \mathcal {V}_{\textrm{sub}} = \mathcal {V}_p \cup \left\{ m \in \mathcal {V}_m \mid \exists p \in \mathcal {V}_p : (m, p) \in \textstyle \bigcup _{q \in \mathcal {V}_p} \mathcal {E}_q \right\} \\ 4.&\ \text {Edge set: } \mathcal {E}_{\textrm{sub}} = \textstyle \bigcup _{p \in \mathcal {V}_p} \mathcal {E}_p \end{aligned}$$

where $\textrm{rank}_p(e)$ denotes the rank of edge $e$ when sorting all edges incident to $p$ by $\textrm{score}(e)$ in descending order. Define the dynamic adjacency matrix generation function:

22 $$\begin{aligned} E_{MHC-peptide} = \text {CrossAttn}(\textbf{H}_m, X_{PB}) \end{aligned}$$

23 $$\begin{aligned} e^{ij} = \frac{\exp \left( \text {LeakyReLU}\left( a^T\left[ \textbf{W}\textbf{h}_m^{(i)} \Vert \textbf{W}\textbf{h}_p^{(j)}\right] \right) \right) }{\sum _{k \in \mathcal {V}_p} \exp \left( \text {LeakyReLU}\left( a^T\left[ \textbf{W}\textbf{h}_m^{(i)} \Vert \textbf{W}\textbf{h}_p^{(k)}\right] \right) \right) } \end{aligned}$$

Based on the edge weights, we construct two adjacency matrices. For each peptide node $\mathcal {P} \subseteq \mathcal {V}_p$, select the top-k MHC nodes, that is, to get the induced subgraph$\mathcal {G}_{\text {sub}} = (\mathcal {V}_{\text {sub}}, \mathcal {E}_{\text {sub}})$, where $\mathcal {V}_{\text {sub}} = \mathcal {P} \cup \left\{ m \in \mathcal {V}_m \mid \exists p \in \mathcal {P}: (m, p) \in \mathcal {E}_{\text {sel}} \right\}$, $\mathcal {E}_{\text {sub}} = \bigcup _{p_j \in \mathcal {P}} \left\{ (m, p_j) \mid m \in \text {Top}_k(p_j) \right\}$. To be specific:

24 $$\begin{aligned} \begin{aligned} \textbf{A}^{ij}&= {\left\{ \begin{array}{ll} \tau (m_i, p_j) & \text {if } m_i \in \text {Top}_k(p_j) \\ 0 & \text {otherwise} \end{array}\right. } \\ \end{aligned} \end{aligned}$$

25 $$\begin{aligned} \text {Top}_k(p_j) = \underset{m \in \mathcal {N}(p_j)}{\text {arg top-k}} \quad \tau (m, p_j) \end{aligned}$$

Adjacency matrix from peptide to MHC and MHC to peptide defined as: $\textbf{A}_{mp} \in \mathbb {R}^{|\mathcal {V}_m| \times |\mathcal {V}_p|} \quad \text {(MHC-peptide)}$, $\textbf{A}_{pm} \in \mathbb {R}^{|\mathcal {V}_p| \times |\mathcal {V}_m|} \quad \text {(peptide-MHC)}$. We define the link relationship as $A=k(A_{pm}+ A_{mp})$, $k=0.5$.

#### GCN module

We use GCN to extract the two types of relationship graph respectively. For $\text {(MHC-peptide)}$ and $\text {(peptide-MHC)}$ graph, we have:

26 $$\begin{aligned} \begin{aligned}&\text {GCN}_{mp}(\textbf{H}_m, X_{PB}) = \sigma \left( \mathbf {\hat{D}}_{mp}^{-1/2} \textbf{A}_{mp} \mathbf {\hat{D}}_{pm}^{-1/2} \textbf{H}_{mp} \mathbf {\Theta }_m \right) \\&\text {GCN}_{pm}(\textbf{H}_m, X_{PB}) = \sigma \left( \mathbf {\hat{D}}_{pm}^{-1/2} \textbf{A}_{pm} \mathbf {\hat{D}}_{mp}^{-1/2} \textbf{H}_{pm} \mathbf {\Theta }_p \right) \end{aligned} \end{aligned}$$

where $\mathbf {\hat{D}}_{mp,pm}$ is degree matrix, $\mathbf {\Theta }$ is the parameter and $\sigma$ is the activation function. The specific calculation is as follows:

27 $$\begin{aligned} \begin{aligned}&\mathbf {\hat{D}}_{mp} = \text {diag}(\sum _j \textbf{A}_{mp}^{ij})\\&\mathbf {\hat{D}}_{pm} = \text {diag}(\sum _i \textbf{A}_{pm}^{ji}) \\ \end{aligned} \end{aligned}$$

The two subgraphs represent the graph aggregation relationships under different paths ($\text {(MHC-peptide)}$ and $\text {(peptide-MHC)}$). Further, we use a weighted approach to fuse these two parts of information to obtain the final result.

28 $$\begin{aligned} \begin{aligned} \textbf{Z}_{mp}&= \text {GCN}_{mp}(\textbf{H}_m, \textbf{H}_p) \\ \textbf{Z}_{pm}&= \text {GCN}_{pm}(\textbf{H}_m, \textbf{H}_p) \\ \textbf{Z}_{\text {final}}&= \alpha \cdot \textbf{Z}_{mp} + \beta \cdot \textbf{Z}_{pm} \\ \end{aligned} \end{aligned}$$

$$\alpha $$, $$\beta = 1 - \alpha $$ are parameters

### Contrastive learning-based multi-feature module

The sequence features are based on the amino acid level and the structural feature is based on the spatial structure of atoms, direct fusion risks modality misalignment. We bridge this gap through contrastive learning, enforcing consistency between sequence and structural embeddings [29]. First, we map the two representations to the contrastive learning space through a projection head:

29 $$\begin{aligned} \begin{aligned}&\quad \textbf{H}_g = \text {LayerNorm}(\text {ReLU}(\textbf{Z}_{final} \textbf{W}_g + \textbf{b}_g))\\&\quad \textbf{H}_s = \text {LayerNorm}(\text {ReLU}(\textbf{S}_{final} \textbf{W}_s + \textbf{b}_s)) \\ \end{aligned} \end{aligned}$$

Here, $\textbf{W}_g$, $\textbf{b}_g$, $\textbf{W}_s$, and $\textbf{b}_s$ is the is the trainable parameters within the linear layer.The two parts of features are ultimately fused through the adaptive weight module by the linear layer:

30 $$\begin{aligned} \textbf{z}^{(i)} = Linear(\textbf{z}_g^{(i)}, \textbf{z}_s^{(i)}) \end{aligned}$$

### Loss function

The representation of the same group of MHC-peptides were taken as positive sample pairs, denote as $\mathcal {P} = \{(i,i) \mid i \in \mathcal {B}\}$, and two characterizations of different MHC-peptides were taken as negative sample pairs, denote as $\mathcal {N} = \{(i,j) \mid i,j \in \mathcal {B}, i \ne j\}$.

31 $$\begin{aligned} \begin{aligned} \mathcal {L}_{\text {cont}} =&\frac{1}{|\mathcal {P}| + |\mathcal {N}|}[ \sum _{(i,i) \in \mathcal {P}} 1 - \textbf{S}_{ii} \\&+ \sum _{(i,j) \in \mathcal {N}} \max (0, \textbf{S}_{ij} - \text {margin})]\\ \end{aligned} \end{aligned}$$

where $\textbf{S}$ is the distance matrix, calculated by cosine similarity denoted as:

32 $$\begin{aligned} \begin{aligned} \textbf{S}_{ij}&= \frac{1}{\tau } \left\langle \hat{\textbf{h}}_g^{(i)}, \hat{\textbf{h}}_s^{(j)} \right\rangle = \frac{1}{\tau } (\hat{\textbf{h}}_g^{(i)})^\top \hat{\textbf{h}}_s^{(j)} \end{aligned} \end{aligned}$$

Our final objective function integrates two complementary loss components, including supervised regression loss and contrastive alignment loss:

33 $$\begin{aligned} L_{total}=\lambda L_{reg}+\beta L_{cont} \end{aligned}$$

where $L_{reg}$ is calculated by Mean squared error (MSE) between ground-truth binding affinities and predicted values:

34 $$\begin{aligned} L_{reg}=\frac{1}{N}\sum \limits _{i = 1}^N {{{({{\hat{z}}_i} - {z_i})}^2}} \end{aligned}$$

**Fig. 2:**
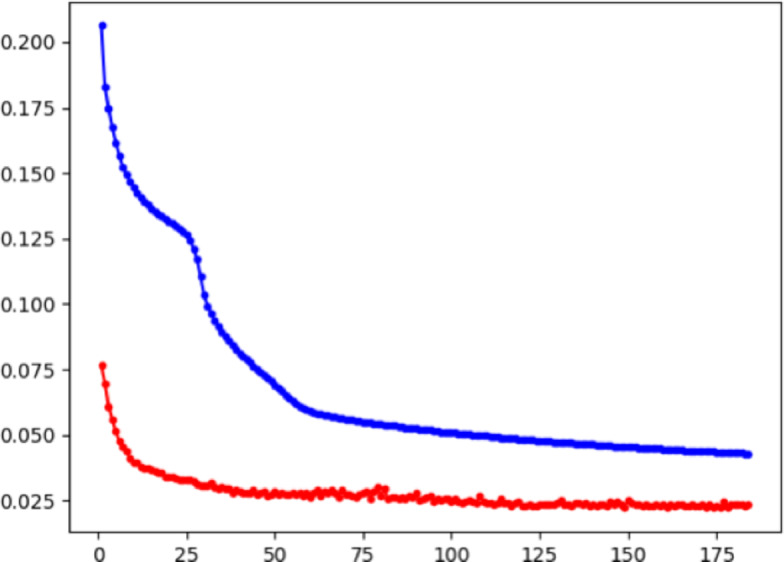
5-CV Training Loss for CMHS in training process. Red line indicate the val loss, blue line is the train loss

## Result

### Datasets

We used the experimentally verified dataset downloaded from Immune Epitope Database(IEDB), which is the most widely used binding affinity (BA) dataset. The dataset covered 80 MHC alleles (43 Human and 12 non-human) with peptides of length 8, 9, 10, 11 for all alleles, and affinity data is given as IC50 values [7, 8, 13]. We further expand MHC data by downloading the HLA-C MHC molecule data and other animal MHC data, including chimpanzee, mouse, and rat, from the Ontobee website, which contains most complete MHC alleles data (https://www.iedb.org/database_export_v3.php).

The peptide-MHC binding affinities are represented by the IC50 values in nM units. We also test the model on the 40 latest weekly benchmark datasets (2021.1.1−2022.11.24), to verify the generality of our model. The datasets are also from IEDB (http://tools.iedb.org/auto_bench/mhci/weekly/). In order to retain the information of the original sequence as much as possible and avoid interference of excessive redundancy, 300 units of amino acids were selected as the final sequence length of MHC I.

### Training set

To evaluate the performance of our CMHS model, we used Pearson Correlation Coefficient (PCC) and Spearmans rank correlation coefficient (SRCC) between predicted and measured binding affinities.

35 $$\begin{aligned} \begin{aligned} \textit{SRCC} = 1 - \frac{6 \sum _{i=1}^{n} d_i^2}{n(n^2 - 1)} \quad \\ \text {where} \quad d_i = \operatorname {rank}(x_i) - \operatorname {rank}(y_i) \end{aligned} \end{aligned}$$

36 $$\begin{aligned} \textit{PCC} = \frac{\sum _{i=1}^{n} (x_i - \bar{x})(y_i - \bar{y})}{\sqrt{\sum _{i=1}^{n} (x_i - \bar{x})^2} \cdot \sqrt{\sum _{i=1}^{n} (y_i - \bar{y})^2}} \end{aligned}$$

Specifically, we use the a threshold of 500 nM to convert IC50 values into one or zero labels. Undering this setting, we used Area Under the Receiver Operating Characteristic Curve (AUROC), Accuray (ACC) as evaluation metrics. PCC measures linear correlation between predicted and true values defined as:

37 $$\begin{aligned} \begin{aligned} \textit{AUC} = \int _{0}^{1} \textit{TPR}(f) \cdot \left| \frac{d\textit{FPR}(f)}{df} \right| df \\ \text {with} \quad \textit{TPR} = \frac{TP}{TP+FN}, \quad \textit{FPR} = \frac{FP}{FP+TN} \end{aligned} \end{aligned}$$

38 $$\begin{aligned} \textit{ACC} = \frac{TP + TN}{TP + TN + FP + FN} \end{aligned}$$

Due to equipment limitations, we adopted performance benchmarks from literature to enable unbiased comparisons. To ensure robust evaluation, we divided the dataset into a training set (80$\%$) and a validation set (20$\%$). A separate test dataset, excluded from model training, was reserved solely for final performance testing. The training dataset underwent 5-fold cross-validation (5-CV) to optimize and validate the model. We use the AdamW optimizer. The epoch is selected as 200 based on the process of the loss during the training Fig. 2. We compared these results with some popular and state-of-the-art methods, including MHCflurry, NetMHCpan 4.1, ANN 4.0, ACME.

- ANN 4.0: Gapped sequence alignment using artificial neural networks for MHC-I and peptide binding affinity prediction [30].
- MHCflurry: A PSSM-based method that predicts binding affinity using sequence similarity to characterized alleles [31].
- NetMHCpan 3.0: An artificial neural network-based method that integratesmass spectrometry-eluted ligands for MHC class I molecules, supporting peptides of length 8-11 [8].
- NetMHCpan 4.1: An artificial neural network-based method that integrates quantitative binding data and mass spectrometry-eluted ligands for a wide range of MHC class I molecules, supporting peptides of length 8-11 [10].
- ACME: Attention-based Convolutional neural networks for MHC Epitope binding prediction model [13].
- RPEMHC: A deep learning-based residue-residue pair encoding method for MHC-peptides affinity prediction [18].

### 5-CV result on the IEDB MHC class I binding affinity dataset

We use $5-$ CV to train the model, with the loss curve is shown in the Fig. 2, on the IEDB dataset on different length of peptides, including 8-mer, 9-mer, 10-mer and 11-mer. We also compared the performance of our model and state-of-the-art methods to shows the efficiency of our model.

**Fig. 3:**
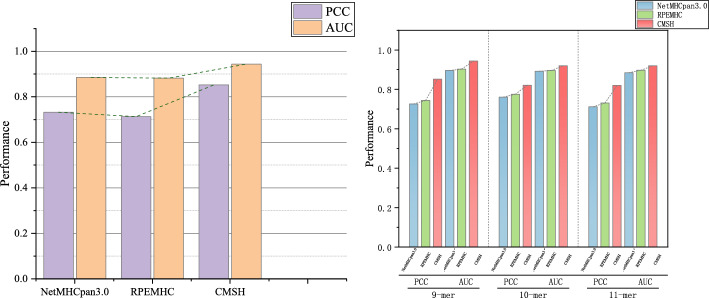
5-CV Training result for CMHS. Lift figure shows 5-CV Training Performances for CMHS on IEDB comparing to SOTA Model. Right figure shows performances for CMHS and SOTA on different length of peptides

The average performance comparison between SOTA and CMHS for different peptide lengths is shown in Fig. 3. For PCC, CMHS achieves 0.8526, outperforming ACME (0.81837) by $4.1\%$ and significantly exceeding NetMHCpan3.0 (0.73233) and RPEMHC (0.7132); For AUROC, CMHS reaches 0.9436, $0.6\%$ higher than ACME (0.9353) with enhanced robustness for all peptides; For ACC, CMHS sets a new record at 0.8911, $1.2\%$ above ACME (0.89077).

Also, we give the result of testing on the IEDB test dataset, demonstrates that CMHS achieves favorable performance across varying peptide lengths, including 9, 10, 11-mer in Fig. 3 right. For 9-mer predictions, CMHS achives a PCC of 0.8526 and AUC of 0.9436, outperforming NetMHCpan3.0: PCC of 0.726, AUC of 0.896) and RPEMHC: PCC of 0.744, AUC of 0.903. With longer peptides, CMHS maintains robust results: 10-mer PCC reaches 0.8215, which has 5.6−6.0 percentage points higher than baselines, while 11-mer AUC sustains at 0.9201 while 0.885 for NetMHCpan3.0. These observations indicate that the CMHS exhibits improved adaptability to diverse peptide-length.

**Fig. 4:**
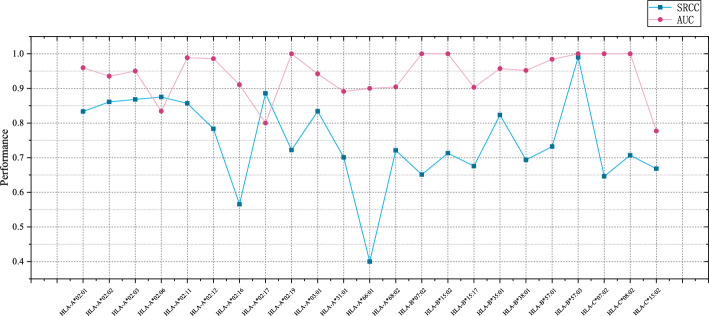
5-CV Training result of performances for CMHS on different MHC alleles

### Comparsion of baselines

Additional testing across 16 alleles for 9-10 mer peptides indicates that CMHS achieves relatively favorable predictive performance in Fig. 4. As shown in Table 1, CMHS attains a PCC value of 0.753, showing improvement over existing methods and exceeding the second-best performer ACME of 0.666 by $8.7\%$. For AUC metrics, CMHS reaches 0.93, outperforming other benchmark models such as NetMHCpan4.0 of 0.815 and SMMPMBEC of 0.824. It is noteworthy that conventional approaches like PickPocket PCC of 0.528 and ANN4.0 with PCC of 0.559 demonstrate relatively limited efficacy in multi-allele dataset.

**Fig. 5:**
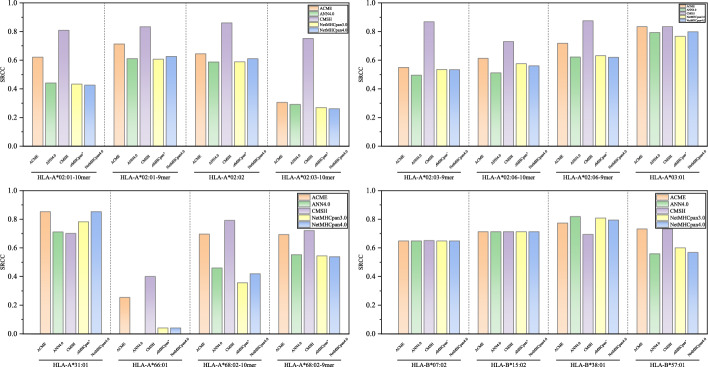
Testing result for CMHS. SRCC Performances for CMHS on IEDB comparing with SOTA Model. Purple line corresponding to the CMHS

**Fig. 6:**
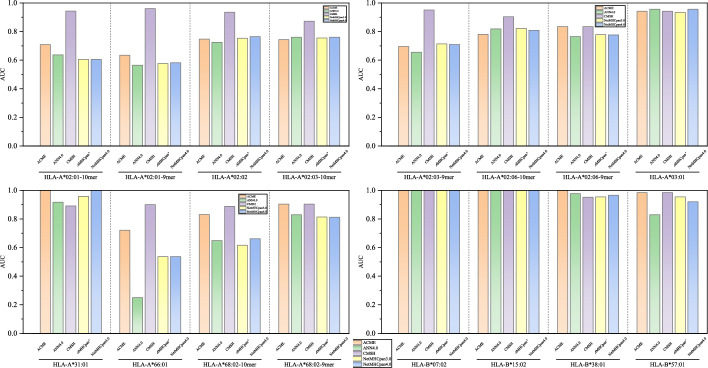
Testing result for CMHS. AUC Performances for CMHS on IEDB comparing with SOTA Model. Purple line corresponding to the CMHS

Besides, detailed testing across 16 alleles indicates that CMHS delivers relatively consistent predictive performance for SRCC shown in Fig. 5, AUC shown in Fig. 6. Taking HLA-A*02:01 as an example, for 10-mer predictions, CMHS achieves an SRCC of $80.9\%$, representing an 18.8 percentage point improvement over ACME eith $62.1\%$. In 9-mer scenarios, its AUC $95.96\%$ exceeds NetMHCpan4.0 $58.2\%$ by 37.76 percentage points. For highly polymorphic HLA-A*02:03, CMHS attains $75.19\%$ SRCC on 10-mers, outperforming baseline methods by approximately 45 percentage points. Notably, on data-scarce HLA-A*66:01, CMHS achieves $40\%$ SRCC percentage points higher than the second-best method, while conventional approaches like ANN4.0 yield negative correlation $-28.7\%$. Although performance converges on prevalent alleles like HLA-B*07:02, CMHS maintains comparatively favorable stability across most complex scenarios.

**Table 1:** Overall performance of CMHS and baseline methods on MHC-peptide binding prediction

Name	Performance	
	SRCC	AUC
ANN4.0	0.559	0.784
IEDB Consensus [32]	0.569	0.8
NetMHCcons [33]	0.595	0.813
NetMHCpan 3.0	0.59	0.81
NetMHCpan 4.0	0.588	0.815
PickPocket [34]	0.528	0.811
SMM [35]	0.578	0.804
SMMPMBEC [36]	0.605	0.824
ACME [13]	0.666	0.854
CMHS(Ours)	0.753	0.93

### Ablation of CMHS

We introduce ablation experiment to shows the contribution of each module. We divided into two parts, including sequence-only: remove all the module of graph; and graph-only: remove the sequence module, including LLMs and CNN-extract and contrast learning. The result is shown in Table 2.

**Table 2:** Performance comparison of CMHS and its variants under different ablation settings

Ablation	Performance	
	SRCC	ACC
CMHS	0.813	0.941
CMHS -without structure	0.7403	0.932
CMHS -without squence	0.4503	0.7658
CMHS -without LoRA	0.796	0.834
CMHS -without contrastive	0.665	0.873
CMHS -without noise layer	0.73	0.889

The ablation study results is shown in Table 2. The full CMHS model achieves best overall performance, with SRCC reaching $81.3\%$ and AUC at $94.1\%$, demonstrating the effectiveness of the proposed design.

Removing the structural representation leads to SRCC degradation drops from $81.3\%$ to$74\%$, indicating that MHC structural modeling plays an important role in capturing spatial constraints. In contrast, removing the sequence-based representation results in a much more SRCC performance drop with $45\%$, shown that sequence information remains a fundamental determinant of binding affinity.

The exclusion of LoRA-based fine-tuning causes a moderate decline in performance SRCC $79.6\%$, ACC $83.4\%$), suggesting that parameter-efficient adaptation of the ESM improves feature expressiveness while avoiding overfitting. This result supports the use of LoRA as an effective lightweight fine-tuning strategy within CMHS.

When contrastive learning is removed, the SRCC decreases to $66.5\%$, indicating that contrastive objectives are essential for aligning multimodal representations and enhancing the discriminability between high- and low-affinity peptide-MHC pairs.

Finally, removing the noise-aware layer also leads to a clear performance drop with SRCC $73.0\%$, confirming that modeling structural uncertainty contributes to more robust affinity prediction, especially in the presence of noisy or predicted structures.

Overall, these ablation results demonstrate that each component of CMHS contributes positively to the final performance, and that the integration of sequence, structure, contrastive learning, LoRA-based adaptation, and noise modeling is critical for achieving optimal predictive accuracy.

## Discussion

In this study, the CMHS provide a framework for MHC-peptide binding prediction by integrating hierarchical graph representation learning, biophysical noise modeling, and LLM-based sequence representations. CMHS decoupling intra-MHC conformational learning from peptide-MHC binding dynamics, together with effective multimodal feature fusion, substantially enhances prediction performance and addresses several limitations of existing approaches. Compared with pseudo sequence based methods, CMHS preserves the spatial topology critical for allosteric regulation, such as long-range communication between the $\alpha$1 helix and $\beta$2-microglobulin in HLA-I molecules, whereas pseudo-sequences tend to fragment these structural pathways. In addition, the proposed B-factor-guided noise-aware layer explicitly models structural uncertainty, enabling the network to account for peptide-induced conformational flexibility. For graph neural network-based approaches, the two-stage hierarchical design mitigates the over-smoothing problem by first stabilizing global MHC structural coherence and subsequently modeling local binding-induced perturbations. Extensive external evaluations demonstrate that CMHS consistently outperforms state-of-the-art methods, including NetMHCpan, MHCflurry, and ACME, achieving superior accuracy and stability.

Our methods involves the introduction of hierarchical graph networks and multimodal feature inevitably increases computational complexity and training time compared with sequence-only methods, which may limit large-scale or real-time applications.

## Data Availability

Data was deposited into the IEDB and are available including the training MHC alleles datasets downloaded from https://www.iedb.org/database_export_v3.php, the weekly datasets downloaded from http://tools.iedb.org/auto_bench/mhci/weekly/. All externally submitted data are from NIH epitope contracts whose projects undergo ethical screening prior to data collection, especially when human or live specimens are in question. Therefore, there is no further assessment to be done by the IEDB.
